# Supplementary material for: Burial leakage: A human accustomed groundwater contaminant sources and health hazards study near cemeteries in Benin City, Nigeria
Source: PLoS One. 2023 Dec 14;18(12):e0292008. doi: 10.1371/journal.pone.0292008 (PMC10721053; doi:10.1371/journal.pone.0292008)
Supplement: Attachment — Submitted filename: Response to Reviewers.docx [file pone.0292008.s002.docx]

**Response to Editor and Reviewers**

Journal requirements:

When submitting your revision, we need you to address these additional requirements.

1. Please ensure that your manuscript meets PLOS ONE's style requirements, including those for file naming. The PLOS ONE style templates can be found at

<https://journals.plos.org/plosone/s/file?id=wjVg/PLOSOne_formatting_sample_main_body.pdf> and

<https://journals.plos.org/plosone/s/file?id=ba62/PLOSOne_formatting_sample_title_authors_affiliations.pdf>

We have now styled the manuscript according to PLOS ONE style

2. In your Methods section, please provide additional information regarding the permits you obtained for the work. Please ensure you have included the full name of the authority that approved the field site access and, if no permits were required, a brief statement explaining why.

We have addressed this in the “Ethics statement”. We wrote “Permission to access and collect groundwater samples from boreholes in the privately-owned houses was granted verbally by all landlords in the study area”. The landlords would however want to remain anonymous.

3. We noticed you have some minor occurrence of overlapping text with the following previous publication(s), which needs to be addressed:

Groundwater pollution by trace metals and human health risk assessment in central west part of Bangladesh - <https://doi.org/10.1016/j.gsd.2019.100219>

Targeting low-arsenic aquifers in Matlab Upazila, Southeastern Bangladesh - <http://dx.doi.org/10.1016/j.scitotenv.2006.06.028>

In your revision ensure you cite all your sources (including your own works), and quote or rephrase any duplicated text outside the methods section. Further consideration is dependent on these concerns being addressed.

We have largely rephrased all the overlapping text especially the two articles stated above among other articles.

4. Thank you for stating the following financial disclosure:

“NO - Include this sentence at the end of your statement: The funders had no role in study design, data collection and analysis, decision to publish, or preparation of the manuscript.”

I cannot see financial disclosure section on the dashboard.

Hence we have now added this statement to the manuscript: “The funders had no role in study design, data collection and analysis, decision to publish, or preparation of the manuscript”.

At this time, please address the following queries:

a) Please clarify the sources of funding (financial or material support) for your study. List the grants or organizations that supported your study, including funding received from your institution.

b) State what role the funders took in the study. If the funders had no role in your study, please state: “The funders had no role in study design, data collection and analysis, decision to publish, or preparation of the manuscript.”

c) If any authors received a salary from any of your funders, please state which authors and which funders.

d) If you did not receive any funding for this study, please state: “The authors received no specific funding for this work.

This is the appropriate category

We cannot see the Financial disclosure section on the submission portal.

5. Please amend either the abstract on the online submission form (via Edit Submission) or the abstract in the manuscript so that they are identical.

Attended to

6. We note that Figure 1 in your submission contain [map/satellite] images which may be copyrighted. All PLOS content is published under the Creative Commons Attribution License (CC BY 4.0), which means that the manuscript, images, and Supporting Information files will be freely available online, and any third party is permitted to access, download, copy, distribute, and use these materials in any way, even commercially, with proper attribution. For these reasons, we cannot publish previously copyrighted maps or satellite images created using proprietary data, such as Google software (Google Maps, Street View, and Earth). For more information, see our copyright guidelines: <http://journals.plos.org/plosone/s/licenses-and-copyright>.

We require you to either (1) present written permission from the copyright holder to publish these figures specifically under the CC BY 4.0 license, or (2) remove the figures from your submission:

a. You may seek permission from the original copyright holder of Figure 1 to publish the content specifically under the CC BY 4.0 license.

We recommend that you contact the original copyright holder with the Content Permission Form (<http://journals.plos.org/plosone/s/file?id=7c09/content-permission-form.pdf>) and the following text:

“I request permission for the open-access journal PLOS ONE to publish XXX under the Creative Commons Attribution License (CCAL) CC BY 4.0 (<http://creativecommons.org/licenses/by/4.0/>). Please be aware that this license allows unrestricted use and distribution, even commercially, by third parties. Please reply and provide explicit written permission to publish XXX under a CC BY license and complete the attached form.”

Please upload the completed Content Permission Form or other proof of granted permissions as an "Other" file with your submission.

In the figure caption of the copyrighted figure, please include the following text: “Reprinted from [ref] under a CC BY license, with permission from [name of publisher], original copyright [original copyright year].”

b. If you are unable to obtain permission from the original copyright holder to publish these figures under the CC BY 4.0 license or if the copyright holder’s requirements are incompatible with the CC BY 4.0 license, please either i) remove the figure or ii) supply a replacement figure that complies with the CC BY 4.0 license. Please check copyright information on all replacement figures and update the figure caption with source information. If applicable, please specify in the figure caption text when a figure is similar but not identical to the original image and is therefore for illustrative purposes only.

The following resources for replacing copyrighted map figures may be helpful:

USGS National Map Viewer (public domain): <http://viewer.nationalmap.gov/viewer/>

The Gateway to Astronaut Photography of Earth (public domain): <http://eol.jsc.nasa.gov/sseop/clickmap/>

Maps at the CIA (public domain): <https://www.cia.gov/library/publications/the-world-factbook/index.html> and <https://www.cia.gov/library/publications/cia-maps-publications/index.html>

NASA Earth Observatory (public domain): <http://earthobservatory.nasa.gov/>

Landsat: <http://landsat.visibleearth.nasa.gov/>

USGS EROS (Earth Resources Observatory and Science (EROS) Center) (public domain): [http://eros.usgs.gov/#](http://eros.usgs.gov/)

Natural Earth (public domain): <http://www.naturalearthdata.com/>

We have now deleted Figure 1

7. Please upload a copy of Supporting Information Table S1 which you refer to in your text on page 17.

We have now uploaded a copy of the supporting information Table S1

8. In your Data Availability statement, you have not specified where the minimal data set underlying the results described in your manuscript can be found. PLOS defines a study's minimal data set as the underlying data used to reach the conclusions drawn in the manuscript and any additional data required to replicate the reported study findings in their entirety. All PLOS journals require that the minimal data set be made fully available. For more information about our data policy, please see <http://journals.plos.org/plosone/s/data-availability>.
Upon re-submitting your revised manuscript, please upload your study’s minimal underlying data set as either Supporting Information files or to a stable, public repository and include the relevant URLs, DOIs, or accession numbers within your revised cover letter. For a list of acceptable repositories, please see <http://journals.plos.org/plosone/s/data-availability#loc-recommended-repositories>. Any potentially identifying patient information must be fully anonymized.
Important: If there are ethical or legal restrictions to sharing your data publicly, please explain these restrictions in detail. Please see our guidelines for more information on what we consider unacceptable restrictions to publicly sharing data: <http://journals.plos.org/plosone/s/data-availability#loc-unacceptable-data-access-restrictions>. Note that it is not acceptable for the authors to be the sole named individuals responsible for ensuring data access.
We will update your Data Availability statement to reflect the information you provide in your cover letter.

We have now uploaded a copy of the supporting information detailing the collected datasets.

Additional Editor Comments:

The authors have improved the manuscript, but not thoroughly enough.
There is a lot of information, but the clarity, coherence, and synthesis are still insufficient, as the reviewers pointed out.

Vey many thanks for your comments; we have now substantially improved the clarity, coherence, synthesis and readability of the manuscript following the Reviewers and Editor’s suggestions and comments.

Reviewer #1: Comments:
1. “Ethics statement” title in “Materials and Methods” should be transferred to the end of conclusion

The Ethics statement have been transferred to the end of conclusion.

2. Add parentheses for “(Fig. 1: the map was plotted using QGIS Desktop 3.16.3 .”

On the recommendation of the journal, we have now deleted Figure 1

3. Use a uniform format for “mgL-1” or “mgl-1” in the text.

This has been unified throughout the manuscript.

4. In equation 10, use multiplication sign “x” correctly.

Done, thanks you.

5. Use comma before “respectively”.

This has been done throughout the manuscript.

6. Table 4, row 3, columns 11 and 12, “-” is given in red color.

The colour effect has been addressed.

**7.** Add limitations of your study.

We have added limitations of the research study to the manuscript.

**8.** Compare your results with more from literature.

We have done this by comparing with more literature across the globe

9. To improve the paper’s Introduction regarding water quality and metals, the authors can read and use the following papers (if applicable):
I. Distribution, exposure, and human health risk analysis of heavy metals in drinking groundwater of Ghayen County, Iran
II. Protocol for the estimation of drinking water quality index (DWQI) in water resources: Artificial neural network (ANFIS) and Arc-Gis
III. Characteristics, water quality index and human health risk from nitrate and fluoride in Kakhk city and its rural areas, Iran

Thanks for highlighting these publication as they more informative, and we have adopted some of them to improve the introduction

Reviewer #2: Reviewer # comments:
Manuscript number: PONE-D-23-11494
Manuscript title: Burial leakage: a human accustomed groundwater contaminant sources and health hazards study near cemeteries in Benin City, Nigeria

Dear Authors, I have now read and assessed your manuscript with the abovementioned details. The topic of the manuscript is interesting. However, I have some concerns that should be carefully addressed. Please, see my queries/specific comments below.

Many thanks to your comments, they are very invaluable.

1. Please sufficiently justify that the selected parameters would provide signatures of leachates that are peculiar to cemeteries. Check whether it is really essential to emphasize on the impact of cemetery.

We have now sufficiently justified the statement in the revised manuscript.

2**. Please emphasize more on the gaps in knowledge and novelty of the present study in the introduction section.**

We have emphasized more on the gap in knowledge and novelty of our study.

**3.** This statement is unclear: “Several studies affirmed that this formation which follows the Oligocene-Pleistocene era in the continent of Africa at the sub-oceanic is underlain by sedimentary formation of the South Sedimentary Basin.” Please refine to make geologic sense.

We have reworded the statement to make more meaning than before.

**4**. “Benin formation” should be written as “Benin Formation”. The norm is that the word “formation” should be capitalized.

This has been formalised.

5. Please provide sufficient details on the surface hydrology, topography, and hydrogeology. These are very important for a better understanding of the influence of cemetery on the water quality.

We have now provided more details on the surface hydrology, topography, and hydrogeology of our study area.

6. Geologic/hydrogeologic map is missing.

This was not included because it was not among the objectives of our study as “Omorogieva OM, Imasuen O. Litho-stratigraphic and hydrogeological evaluation of groundwater system in parts of Benin Metropolis, Benin City Nigeria: The key to groundwater sustainability. J. Appl. Sci. Environ. Manage. 2018; 22: 275 – 280” have provided the required details in their study.

7. The results and discussion section should be robust. Compare your findings with literature data from other regions.

Attended to as suggested.

**8.** Please refer to these recent articles to improve the literature review, methodology and discussions: <https://doi.org/10.1007/s13201-020-01180-9>; <https://doi.org/10.1080/15275922.2021.2006363>; <https://doi.org/10.1007/s10661-022-09789-w>; <https://doi.org/10.1080/10106049.2022.2034990>; <https://doi.org/10.1016/j.heliyon.2023.e15483>; <https://doi.org/10.1016/j.ecolind.2023.110287>; <https://doi.org/10.1007/s11356-023-26396-5>

Thanks for highlighting these publication as they are more informative, and we have adopted some of them to improve literature review, methodology and discussions

9. The quality of the figures is poor. Please replace them with better versions.

We have now provided improved TIFF image. Please note that the journal may not provide reviewers with high quality image during the revision stage.

10. The novelty and significances of the current study should also be justified in the conclusions.

This has been done.

**11. What are the limitations of the present study? Highlight on them**.

We have added limitations of the research study to the manuscript

**12. Also, discuss the perspectives for future research**.

This has been included to the manuscript.

Thanks.
